# Pyridinium *trans*-di­aqua­bis­[oxalato(2−)-κ^2^ *O* ^1^,*O* ^2^]chromate(III) urea monosolvate

**DOI:** 10.1107/S1600536813026135

**Published:** 2013-09-28

**Authors:** Gouet Bebga, Martin Signé, Justin Nenwa, Mohammed Mbarki, Boniface P. T. Fokwa

**Affiliations:** a Department of Chemistry, Higher Teacher Training College, University of Yaounde I, POB 47, Cameroon; b Departement of Inorganic Chemistry, University of Yaounde I, POB 812 Yaounde, Cameroon; c Institut für Anorganische Chemie, RWTH Aachen, D-52056 Aachen, Germany

## Abstract

The asymmetric unit of the title solvated mol­ecular salt, (C_5_H_6_N)[Cr(C_2_O_4_)_2_(H_2_O)_2_]·CO(NH_2_)_2_, contains half a formula unit. Each component is completed by crystallographic twofold symmetry: in the cation, one C and the N atom lie on the rotation axis; in the anion, the Cr^III^ ion lies on the axis; in the solvent mol­ecule, the C and the O atom lie on the axis. The aqua ligands are in a *trans* disposition in the resulting CrO_6_ octa­hedron. In the crystal, the components are linked by O—H⋯O, N—H⋯O and N—H⋯(O,O) hydrogen bonds, generating a three-dimensional network.

## Related literature

For mol­ecular salts containing the [Cr(C_2_O_4_)_2_(H_2_O)_2_]^−^ anion, see: Bélombé *et al.* (2009 ▶); Nenwa *et al.* (2010 ▶, 2012 ▶); Chérif *et al.* (2011 ▶); Chérif, Zid *et al.* (2012 ▶); Chérif, Abdelhak *et al.* (2012 ▶); Dridi *et al.* (2013 ▶).
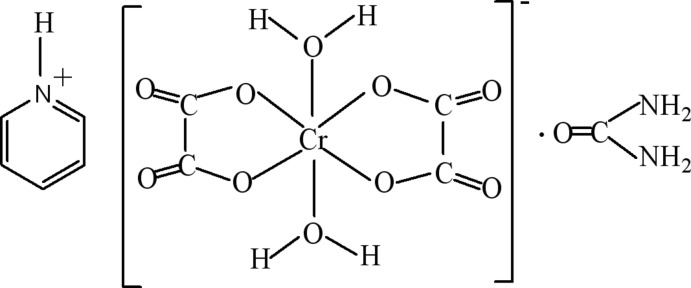


## Experimental

### 

#### Crystal data

(C_5_H_6_N)[Cr(C_2_O_4_)_2_(H_2_O)_2_]·CH_4_N_2_O
*M* *_r_* = 404.24
Monoclinic, 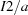

*a* = 7.6456 (7) Å
*b* = 21.4096 (18) Å
*c* = 9.7404 (12) Å
β = 100.278 (1)°
*V* = 1568.8 (3) Å^3^
*Z* = 4
Mo *K*α radiation
μ = 0.80 mm^−1^
*T* = 293 K
0.20 × 0.16 × 0.13 mm

#### Data collection

Bruker APEX CCD diffractometer
Absorption correction: multi-scan (*SADABS*; Bruker, 2004 ▶) *T*_min_ = 0.851, *T*_max_ = 0.935
11770 measured reflections
2343 independent reflections
1980 reflections with *I* > 2σ(*I*)
*R*_int_ = 0.032

#### Refinement

*R*[*F* ^2^ > 2σ(*F* ^2^)] = 0.041
*wR*(*F* ^2^) = 0.111
*S* = 1.10
2343 reflections
132 parameters
5 restraints
H atoms treated by a mixture of independent and constrained refinement
Δρ_max_ = 0.30 e Å^−3^
Δρ_min_ = −0.56 e Å^−3^

### 

Data collection: *SMART* (Bruker, 2004 ▶); cell refinement: *SAINT* (Bruker, 2004 ▶); data reduction: *SAINT*; program(s) used to solve structure: *SHELXS97* (Sheldrick, 2008 ▶); program(s) used to refine structure: *SHELXL97* (Sheldrick, 2008 ▶); molecular graphics: *DIAMOND* (Brandenburg, 2010 ▶); software used to prepare material for publication: *WinGX* (Farrugia, 2012 ▶).

## Supplementary Material

Crystal structure: contains datablock(s) I. DOI: 10.1107/S1600536813026135/hb7141sup1.cif

Structure factors: contains datablock(s) I. DOI: 10.1107/S1600536813026135/hb7141Isup2.hkl

Additional supplementary materials:  crystallographic information; 3D view; checkCIF report

## Figures and Tables

**Table 1:** Selected bond lengths (Å)

Cr4—O2	1.9436 (12)
Cr4—O3	1.9762 (12)
Cr4—O1	1.9955 (14)

**Table 2:** Hydrogen-bond geometry (Å, °)

*D*—H⋯*A*	*D*—H	H⋯*A*	*D*⋯*A*	*D*—H⋯*A*
N1—H1⋯O4^i^	0.86	2.26	2.979 (3)	142
N1—H1⋯O4^ii^	0.86	2.26	2.979 (3)	142
N2—H2*A*⋯O4^iii^	0.82 (2)	2.36 (2)	3.134 (2)	158 (3)
N2—H2*B*⋯O5^iv^	0.79 (2)	2.08 (2)	2.847 (2)	166 (3)
O1—H1*B*⋯O3^iii^	0.81 (2)	1.91 (2)	2.7135 (18)	174 (3)
O1—H1*A*⋯O6	0.81 (2)	1.79 (2)	2.5910 (16)	176 (3)

Symmetry codes: (i) 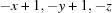; (ii) 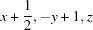; (iii) 
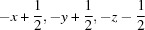
; (iv) 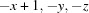.

**Fig. 1.**
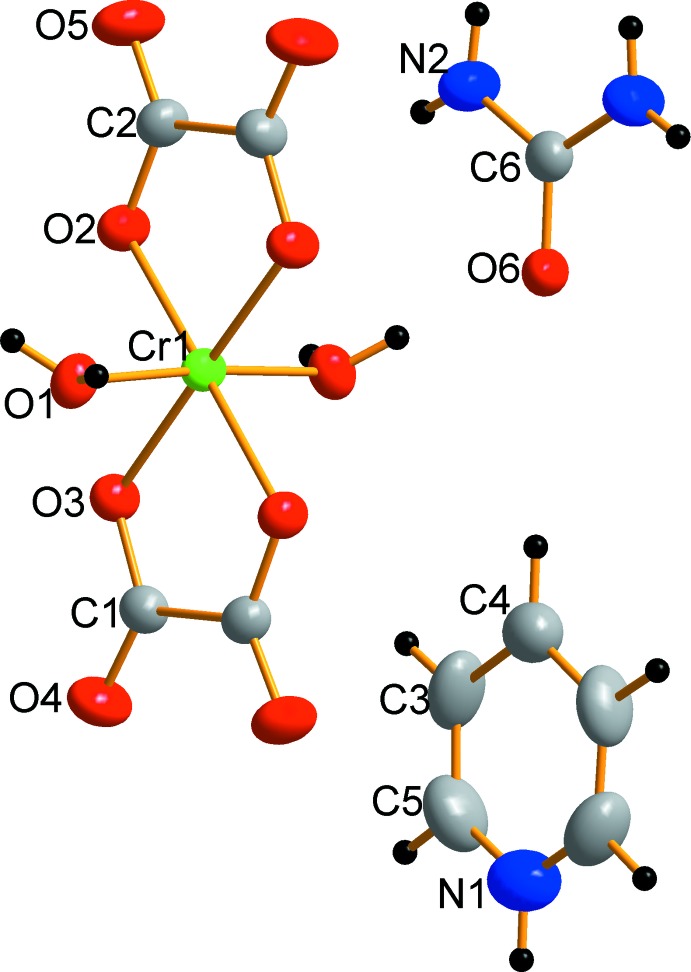
Molecular structure of the title compound with displacement ellipsoids drawn at the 50% probability level.

**Fig. 2.**
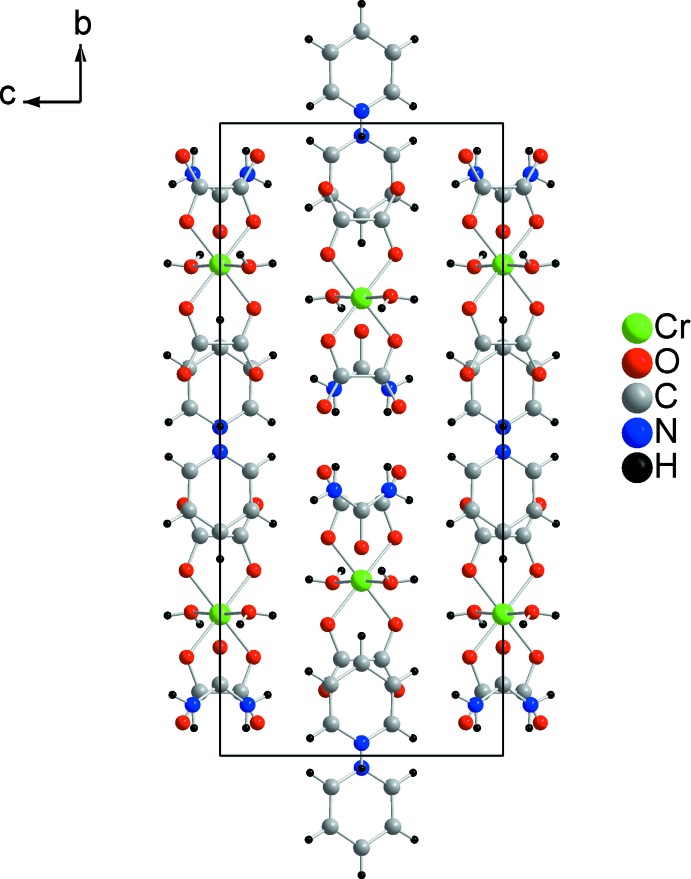
Packing diagram of the title compound, viewed along the a axis, showing its layered structure.

**Fig. 3.**
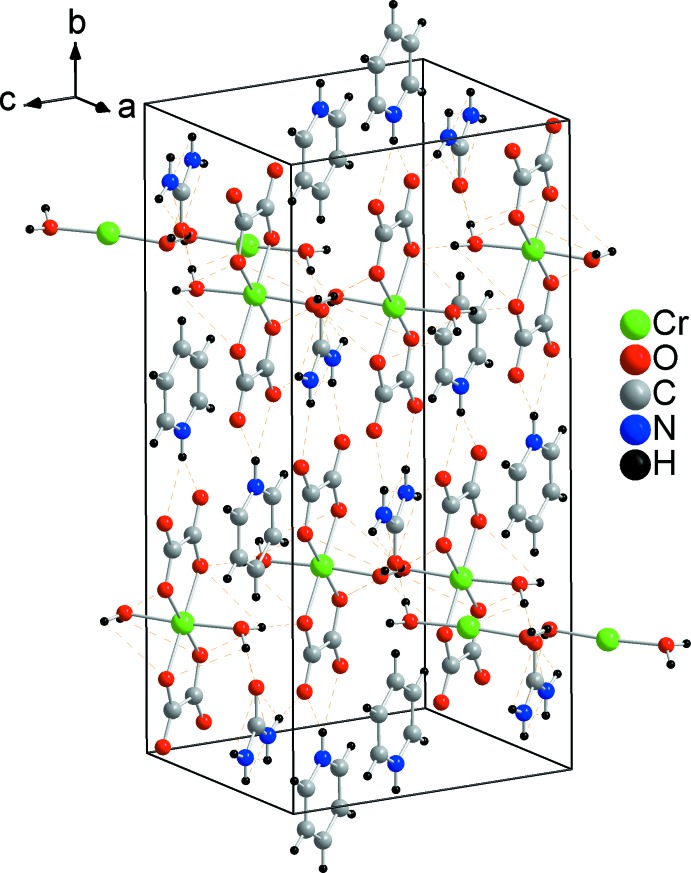
Interconnection of the constituents of the title compound into a three-dimensional network. Hydrogen bonds are highlighted with dashed lines.

## supplementary crystallographic information

### 1. Comment

Recently, the crystal structures of some salts involving organic cations and the complex anion [Cr(C_2_O_4_)_2_(H_2_O)_2_]^-^, have been reported (Bélombé *et al.*,2009; Nenwa *et al.*, 2010; Chérif, Zid*et al.*, 2012; Chérif, Abdelhak *et al.*, 2012; Nenwa, Gouet *et al. 2012*). We now report the structure of the title compound, with the organic cation, pyridinium, the *trans*-diaquabis(oxalate)chromate(III) complex anion and the urea molecule which replaces the fraction of water molecule of crystallization in the previously described structures (Chérif *et al.*, 2011; Chérif, Abdelhak *et al.*,2012; Dridi *et al.*, 2013).

The constituents of the title compound are shown in Fig.1. It appears to be the first member of salts with general formula A_m_[*M*(C_2_O_4_)_2_(H_2_O)_2_].xOC(NH_2_)_2_; where A = organic cation, *M* = metal(II) or metal(III), m = 1 or 2 and *x*≥ 0. The asymmetric unit is formed by a pyridinium cation, a [Cr(C_2_O_4_)_2_(H_2_O)_2_]^–^ anionic complex in *trans*-aqua configuration and one urea molecule. The chromium (III) ion lies on a twofold axis and is six-coordinated in a distorted octahedral geometry defined by four O atoms from two chelating bidendate oxalate anions in the equatorial plane and by two O atoms from two apical aqua ligands. The equatorial Cr–O_(oxalate)_ distances, 1.9435 (13) Å and 1.9762 (13) Å, are slightly shorter than the axial Cr–O_(water)_ one [1.9955 (15) Å]. These bond distances are similar to those observed in homologous complex salts (Bélombé *et al.*, 2009; Nenwa *et al.*, 2010; Chérif *et al.*, 2011; Chérif, Abdelhak *et al.*, 2012; Chérif, Abdelhak *et al.*, 2012; Nenwa *et al.*, 2012; Dridi *et al.*, 2013).

The crystal structure can be described by a characteristic layered arrangement of the pyridinium cation, (C_5_H_6_N)^+^, the complex anion, [Cr(C_2_O_4_)_2_(H_2_O)_2_]^-^, and the urea molecule (Fig. 2). O–H···O and N–H···O hydrogen bonding interactions link the components (Fig.3).

### 2. Experimental

1 mmol (267 mg) of CrCl_3_·6H_2_O was dissolved in 50 ml of water. The green filtered solution was stirred at 323 K, 2 mmol (253 mg) of oxalic acid, 1 mmol (79.1 mg) of pyridine and 2 mmol (121 mg) of urea were added in successive small portions and stirred for 2 h. The resulting violet solution was left at room temperature; violet prisms were obtained after one week of slow evaporation.

### 3. Refinement

The H atoms of the pyridimium cation were positioned geometrically, with C—H, N—H distances of 0.93 and 0.86 Å respectively, and constrained to ride on their parent atoms, with *U*_iso_(H) = 1.2*U*_eq_(C, N). The urea H atoms were located in a difference Fourier map and freely refined.

### Crystal data

**Table:**

(C_5_H_6_N)[Cr(C_2_O_4_)_2_(H_2_O)_2_]·CH_4_N_2_O	*Z* = 4
*M**_r_* = 404.24	*F*(000) = 828
Monoclinic, *I*2/*a*	*D*_x_ = 1.712 Mg m^−^^3^
Hall symbol: -I 2ya	Mo *K*α radiation, λ = 0.71073 Å
*a* = 7.6456 (7) Å	µ = 0.80 mm^−^^1^
*b* = 21.4096 (18) Å	*T* = 293 K
*c* = 9.7404 (12) Å	Prism, violet
β = 100.278 (1)°	0.20 × 0.16 × 0.13 mm
*V* = 1568.8 (3) Å^3^	

### Data collection

**Table:**

Bruker APEX CCD diffractometer	2343 independent reflections
Radiation source: fine-focus sealed tube	1980 reflections with *I* > 2σ(*I*)
Graphite monochromator	*R*_int_ = 0.032
ω scans	θ_max_ = 30.9°, θ_min_ = 2.3°
Absorption correction: multi-scan (*SADABS*; Bruker, 2004)	*h* = −10→10
*T*_min_ = 0.851, *T*_max_ = 0.935	*k* = −30→30
11770 measured reflections	*l* = −13→14

### Refinement

**Table:**

Refinement on *F*^2^	Primary atom site location: structure-invariant direct methods
Least-squares matrix: full	Secondary atom site location: difference Fourier map
*R*[*F*^2^ > 2σ(*F*^2^)] = 0.041	Hydrogen site location: inferred from neighbouring sites
*wR*(*F*^2^) = 0.111	H atoms treated by a mixture of independent and constrained refinement
*S* = 1.10	*w* = 1/[σ^2^(*F*_o_^2^) + (0.0515*P*)^2^ + 1.3542*P*] where *P* = (*F*_o_^2^ + 2*F*_c_^2^)/3
2343 reflections	(Δ/σ)_max_ < 0.001
132 parameters	Δρ_max_ = 0.30 e Å^−^^3^
5 restraints	Δρ_min_ = −0.56 e Å^−^^3^

### Special details

**Table:**

Geometry. All s.u.'s (except the s.u. in the dihedral angle between two l.s. planes) are estimated using the full covariance matrix. The cell s.u.'s are taken into account individually in the estimation of s.u.'s in distances, angles and torsion angles; correlations between s.u.'s in cell parameters are only used when they are defined by crystal symmetry. An approximate (isotropic) treatment of cell s.u.'s is used for estimating s.u.'s involving l.s. planes.
Refinement. Refinement of *F*^2^ against ALL reflections. The weighted *R*-factor *wR* and goodness of fit *S* are based on *F*^2^, conventional *R*-factors *R* are based on *F*, with *F* set to zero for negative *F*^2^. The threshold expression of *F*^2^ > 2σ(*F*^2^) is used only for calculating *R*-factors(gt) *etc*. and is not relevant to the choice of reflections for refinement. *R*-factors based on *F*^2^ are statistically about twice as large as those based on *F*, and *R*- factors based on ALL data will be even larger.

### Fractional atomic coordinates and isotropic or equivalent isotropic displacement parameters (Å^2^)

**Table:**

	*x*	*y*	*z*	*U*_iso_*/*U*_eq_	
Cr4	0.2500	0.223340 (17)	0.0000	0.02410 (13)	
O1	0.45417 (19)	0.22672 (6)	−0.10198 (14)	0.0315 (3)	
O2	0.35892 (18)	0.15558 (6)	0.11799 (13)	0.0324 (3)	
O3	0.14209 (17)	0.29281 (6)	−0.11952 (12)	0.0288 (3)	
O4	0.1309 (2)	0.39675 (7)	−0.12681 (15)	0.0436 (4)	
O5	0.3604 (2)	0.05182 (7)	0.13176 (16)	0.0498 (4)	
C1	0.1842 (2)	0.34777 (8)	−0.07105 (17)	0.0285 (3)	
C2	0.3134 (3)	0.10049 (8)	0.07197 (18)	0.0319 (4)	
N1	0.7500	0.48039 (15)	0.0000	0.0622 (8)	
H1	0.7500	0.5206	0.0000	0.075*	
C3	0.8444 (3)	0.38759 (15)	0.1108 (3)	0.0576 (7)	
H3	0.9099	0.3668	0.1868	0.069*	
C4	0.7500	0.35421 (17)	0.0000	0.0527 (8)	
H4	0.7500	0.3108	0.0000	0.063*	
C5	0.8402 (4)	0.45021 (15)	0.1071 (3)	0.0594 (7)	
H5	0.9023	0.4727	0.1819	0.071*	
O6	0.7500	0.17120 (9)	0.0000	0.0388 (4)	
N2	0.6418 (3)	0.08022 (9)	−0.0971 (2)	0.0447 (4)	
C6	0.7500	0.11234 (13)	0.0000	0.0326 (5)	
H2A	0.584 (3)	0.0960 (12)	−0.168 (2)	0.055 (8)*	
H2B	0.649 (4)	0.0435 (8)	−0.092 (3)	0.061 (9)*	
H1B	0.434 (3)	0.2215 (11)	−0.1855 (17)	0.049 (8)*	
H1A	0.544 (3)	0.2077 (11)	−0.071 (2)	0.053 (8)*	

### Atomic displacement parameters (Å^2^)

**Table:**

	*U*^11^	*U*^22^	*U*^33^	*U*^12^	*U*^13^	*U*^23^
Cr4	0.0280 (2)	0.0240 (2)	0.01765 (18)	0.000	−0.00312 (13)	0.000
O1	0.0307 (7)	0.0389 (7)	0.0229 (6)	0.0050 (5)	−0.0004 (5)	−0.0009 (5)
O2	0.0385 (7)	0.0280 (6)	0.0259 (6)	0.0010 (5)	−0.0071 (5)	0.0020 (5)
O3	0.0334 (6)	0.0290 (6)	0.0213 (5)	0.0013 (5)	−0.0020 (5)	0.0010 (4)
O4	0.0565 (9)	0.0310 (7)	0.0391 (8)	0.0071 (6)	−0.0026 (7)	0.0067 (6)
O5	0.0713 (11)	0.0278 (7)	0.0433 (8)	0.0053 (7)	−0.0084 (8)	0.0046 (6)
C1	0.0313 (9)	0.0293 (8)	0.0245 (8)	0.0015 (6)	0.0043 (6)	0.0013 (6)
C2	0.0358 (9)	0.0303 (8)	0.0272 (8)	0.0024 (7)	−0.0014 (7)	0.0024 (6)
N1	0.069 (2)	0.0486 (17)	0.071 (2)	0.000	0.0156 (17)	0.000
C3	0.0445 (13)	0.0814 (19)	0.0409 (12)	0.0074 (12)	−0.0084 (10)	0.0166 (12)
C4	0.0477 (19)	0.0471 (18)	0.062 (2)	0.000	0.0057 (16)	0.000
C5	0.0567 (15)	0.0726 (18)	0.0456 (13)	−0.0166 (13)	0.0001 (11)	−0.0139 (12)
O6	0.0303 (9)	0.0295 (9)	0.0514 (12)	0.000	−0.0067 (8)	0.000
N2	0.0520 (11)	0.0336 (9)	0.0434 (10)	−0.0022 (8)	−0.0052 (8)	−0.0043 (8)
C6	0.0291 (12)	0.0338 (12)	0.0346 (12)	0.000	0.0052 (10)	0.000

### Geometric parameters (Å, º)

**Table:**

Cr4—O2^i^	1.9436 (12)	N1—C5^ii^	1.313 (3)
Cr4—O2	1.9436 (12)	N1—C5	1.313 (3)
Cr4—O3^i^	1.9762 (12)	N1—H1	0.8600
Cr4—O3	1.9762 (12)	C3—C5	1.341 (4)
Cr4—O1	1.9955 (14)	C3—C4	1.385 (3)
Cr4—O1^i^	1.9955 (14)	C3—H3	0.9300
O1—H1B	0.808 (16)	C4—C3^ii^	1.385 (3)
O1—H1A	0.808 (16)	C4—H4	0.9300
O2—C2	1.287 (2)	C5—H5	0.9300
O3—C1	1.287 (2)	O6—C6	1.260 (3)
O4—C1	1.217 (2)	N2—C6	1.331 (2)
O5—C2	1.216 (2)	N2—H2A	0.821 (17)
C1—C1^i^	1.559 (3)	N2—H2B	0.788 (17)
C2—C2^i^	1.556 (3)	C6—N2^ii^	1.331 (2)
			
O2^i^—Cr4—O2	83.43 (7)	O3—C1—C1^i^	113.91 (9)
O2^i^—Cr4—O3^i^	179.29 (5)	O5—C2—O2	125.43 (17)
O2—Cr4—O3^i^	97.10 (5)	O5—C2—C2^i^	120.99 (11)
O2^i^—Cr4—O3	97.10 (5)	O2—C2—C2^i^	113.59 (9)
O2—Cr4—O3	179.29 (5)	C5^ii^—N1—C5	121.0 (4)
O3^i^—Cr4—O3	82.36 (7)	C5^ii^—N1—H1	119.5
O2^i^—Cr4—O1	91.34 (6)	C5—N1—H1	119.5
O2—Cr4—O1	91.76 (6)	C5—C3—C4	119.2 (2)
O3^i^—Cr4—O1	89.11 (5)	C5—C3—H3	120.4
O3—Cr4—O1	87.76 (5)	C4—C3—H3	120.4
O2^i^—Cr4—O1^i^	91.76 (6)	C3—C4—C3^ii^	117.9 (4)
O2—Cr4—O1^i^	91.34 (6)	C3—C4—H4	121.1
O3^i^—Cr4—O1^i^	87.76 (5)	C3^ii^—C4—H4	121.1
O3—Cr4—O1^i^	89.11 (5)	N1—C5—C3	121.3 (3)
O1—Cr4—O1^i^	175.84 (8)	N1—C5—H5	119.3
Cr4—O1—H1B	117.8 (19)	C3—C5—H5	119.3
Cr4—O1—H1A	119.1 (19)	C6—N2—H2A	124 (2)
H1B—O1—H1A	108 (2)	C6—N2—H2B	116 (2)
C2—O2—Cr4	114.66 (11)	H2A—N2—H2B	119 (3)
C1—O3—Cr4	114.90 (10)	O6—C6—N2	121.10 (13)
O4—C1—O3	125.58 (16)	O6—C6—N2^ii^	121.10 (13)
O4—C1—C1^i^	120.51 (11)	N2—C6—N2^ii^	117.8 (3)

Symmetry codes: (i) −*x*+1/2, *y*, −*z*; (ii) −*x*+3/2, *y*, −*z*.

### Hydrogen-bond geometry (Å, º)

**Table:**

*D*—H···*A*	*D*—H	H···*A*	*D*···*A*	*D*—H···*A*
N1—H1···O4^iii^	0.86	2.26	2.979 (3)	142
N1—H1···O4^iv^	0.86	2.26	2.979 (3)	142
N2—H2*A*···O4^v^	0.82 (2)	2.36 (2)	3.134 (2)	158 (3)
N2—H2*B*···O5^vi^	0.79 (2)	2.08 (2)	2.847 (2)	166 (3)
O1—H1*B*···O3^v^	0.81 (2)	1.91 (2)	2.7135 (18)	174 (3)
O1—H1*A*···O6	0.81 (2)	1.79 (2)	2.5910 (16)	176 (3)

Symmetry codes: (iii) −*x*+1, −*y*+1, −*z*; (iv) *x*+1/2, −*y*+1, *z*; (v) −*x*+1/2, −*y*+1/2, −*z*−1/2; (vi) −*x*+1, −*y*, −*z*.
